# Orlistat-Associated Gastrointestinal, Hepatobiliary, Pancreatic, and Anorectal Safety Signals: A FAERS Disproportionality and Regulatory Label Concordance Study

**DOI:** 10.3390/ph19091325

**Published:** 2026-08-22

**Authors:** Deniz Öğütmen Koç, İbrahim Sarbay, Melike Mercan Başpınar, Lütfi Mangal, Burcu Eda Arda, Hande Sipahi

**Affiliations:** 1 Department of Gastroenterology, University of Health Sciences, Gaziosmanpaşa Training and Research Hospital, Istanbul 34255, Türkiye; 2 Department of Emergency Medicine, University of Health Sciences, Gaziosmanpaşa Training and Research Hospital, Istanbul 34255, Türkiye; 3 Department of Family Medicine, University of Health Sciences, Gaziosmanpaşa Training and Research Hospital, Istanbul 34255, Türkiye; 4 Department of Pharmaceutical Toxicology, Faculty of Pharmacy, Harran University, Urfa 63300, Türkiye; 5 Department of Pharmaceutical Toxicology, Faculty of Pharmacy, University of Health Sciences, Istanbul 34668, Türkiye

**Keywords:** orlistat, pharmacovigilance, FAERS, disproportionality analysis, NAFLD/MASLD, gastrointestinal adverse events, hepatobiliary events, constipation, cholecystitis, regulatory label concordance

## Abstract

**Background/Objectives**: Orlistat is a gastrointestinal lipase inhibitor used for weight management. Although its established safety profile is largely characterised by fat malabsorption-related gastrointestinal events, post-marketing reports suggest a broader spectrum of adverse events. Given the increasing intersection between obesity pharmacotherapy and the management of non-alcoholic fatty liver disease/metabolic dysfunction-associated steatotic liver disease (NAFLD/MASLD), this study evaluated gastrointestinal, hepatobiliary, pancreatic, and anorectal adverse-event reporting signals associated with orlistat in the U.S. Food and Drug Administration (FDA) Adverse Event Reporting System (FAERS) and assessed concordance with regulatory prescribing information. **Methods**: Quarterly FAERS files from 2004 Q1 through 2026 Q1 were analysed; some retained reports had Initial FDA Received Date (FDA_DT) values dating back to 1999. Descriptive analyses included all orlistat-associated reports, whereas disproportionality analyses were restricted to primary-suspect reports. Sixty-five prespecified Medical Dictionary for Regulatory Activities (MedDRA) preferred terms (PTs) were screened. Reporting odds ratios (RORs), 95% confidence intervals (CIs), and proportional reporting ratios (PRRs) were calculated. A signal was defined as ROR > 2.0, lower bound of the 95% CI > 1.0, and *n* ≥ 3. Two-sided *p* values calculated using Fisher’s exact test on the corresponding 2 × 2 contingency tables were adjusted using the Benjamini–Hochberg false discovery rate procedure to address multiple testing. Signal-positive PTs were cross-referenced with prescribing information from three jurisdictions. **Results**: Overall, 30,454 deduplicated orlistat-associated reports were identified, including 16,684 primary-suspect reports. Thirty-eight of the 65 prespecified MedDRA PTs met the signal criteria, and all remained statistically significant after Benjamini–Hochberg false discovery rate correction (q < 0.05). The strongest signals were rectal discharge (ROR 1175.53; 95% CI 1093.44–1263.78) and steatorrhoea (ROR 1147.99; 95% CI 1058.12–1245.50); change in bowel habit also showed a high ROR (47.32; 95% CI 38.27–58.51). Unlabelled signal-positive PTs included constipation, faeces hard, cholecystitis, and irritable bowel syndrome; change in bowel habit showed jurisdiction-specific labelling discordance. **Conclusions**: Orlistat-associated FAERS signals included both expected fat-malabsorption-related events and additional gastrointestinal, hepatobiliary, pancreatic, and anorectal PTs, several of which were not explicitly represented in the evaluated regulatory labels. These findings reflect disproportionate reporting rather than incidence, absolute risk, or causality and require confirmation in independent clinical or epidemiological data sources. They do not, by themselves, support regulatory labelling changes.

## 1. Introduction

Obesity is a major public health problem associated with type 2 diabetes, cardiovascular disease, non-alcoholic fatty liver disease/metabolic dysfunction-associated steatotic liver disease (NAFLD/MASLD), and certain malignancies [1]. According to the World Health Organization, more than one billion people worldwide were living with obesity in 2022 [2]. Although lifestyle modification remains the cornerstone of treatment, pharmacological therapies may support weight management in appropriate patients [1].

Orlistat is a locally acting gastrointestinal lipase inhibitor used in the treatment of obesity. By inhibiting gastric and pancreatic lipases, it reduces the hydrolysis of dietary triglycerides into absorbable free fatty acids and monoglycerides; consequently, intestinal fat absorption decreases by approximately 30%, and unabsorbed fat is excreted in the stool [1,3]. Its low systemic exposure and primary site of action within the gastrointestinal lumen distinguish orlistat from weight-management drugs with more pronounced systemic metabolic effects [1,3]. However, a recent systematic review and meta-analysis showed that the weight-loss efficacy of orlistat is more limited than that of newer agents such as semaglutide and tirzepatide [4].

The recent U.S. Food and Drug Administration (FDA) approval of Wegovy (semaglutide) for adults with metabolic dysfunction-associated steatohepatitis (MASH) and moderate-to-advanced liver fibrosis underscores the increasing overlap between obesity pharmacotherapy and liver disease management [5]. Although newer anti-obesity agents generally produce greater weight loss, orlistat remains clinically relevant because of its distinct pharmacological and safety profiles [1,3,4]. Potential benefits on hepatic steatosis, liver enzymes, and certain metabolic parameters have also been reported in patients with NAFLD/MASLD [6,7,8]. Therefore, contemporary real-world safety data on orlistat remain relevant in the context of NAFLD/MASLD.

The known safety profile of orlistat is largely characterised by gastrointestinal adverse events related to fat malabsorption. Oily spotting, flatus with discharge, faecal urgency, fatty/oily stool, increased defaecation frequency, and faecal incontinence are among the typical orlistat-associated adverse events [1,3]. Although these events are consistent with the expected pharmacological effect of the drug, they may negatively affect treatment persistence [9]. However, post-marketing reports suggest a broader range of gastrointestinal and related adverse events beyond the classic manifestations of fat malabsorption [10,11,12].

Paradoxical bowel-pattern reports, including constipation and hard stools, have been described despite the expected effects of oily stool and increased defaecation frequency [10]. Hepatobiliary events, pancreatitis, and anorectal complaints have also been reported [11,12]. Although a previous FAERS analysis identified prominent gastrointestinal and hepatobiliary reporting patterns for orlistat [12], a focused preferred-term-level evaluation of gastrointestinal, hepatobiliary, pancreatic, and anorectal events, together with cross-jurisdictional label comparison, remains warranted. Accordingly, the predefined clinical scope encompassed gastrointestinal and anorectal events related to the local gastrointestinal action and fat-malabsorption profile of orlistat, together with hepatobiliary and pancreatic events supported by regulatory prescribing information and previous pharmacovigilance findings [1,3,11,12]. On the basis of this predefined clinical framework, the final list of 65 MedDRA preferred terms was finalised before calculation and interpretation of the disproportionality results.

Spontaneous adverse-event reporting systems are important resources for post-marketing signal detection. FAERS is a large-scale pharmacovigilance database widely used for the assessment of post-marketing adverse-event reports [13]. Disproportionality analysis enables evaluation of whether specific drug–adverse event pairs are reported more frequently than expected relative to other drugs [14]. Because FAERS lacks reliable exposure denominators and is subject to multiple reporting biases, disproportionality signals do not establish incidence, clinical risk, or causality and should be interpreted as hypothesis-generating findings [13,14,15].

This study evaluated gastrointestinal, hepatobiliary, pancreatic, and anorectal adverse-event reporting signals associated with orlistat in FAERS from 2004 Q1 through 2026 Q1 using disproportionality analysis. Signal-positive PTs were also compared with prescribing information from Türkiye, the European Union, and the United States to characterise terminology and coverage differences across jurisdictions.

## 2. Results

### 2.1. Overview of the FAERS Dataset

After deduplication, 30,454 orlistat-associated reports were retained for descriptive analyses, spanning 1999 through 2026 Q1. Of these, 16,684 reports in which orlistat was designated as the primary-suspect drug formed the disproportionality-analysis population. The comparator dataset comprised 15,680,472 deduplicated reports involving all other drugs. The United States accounted for 21,079 reports (69.2%), followed by Great Britain (*n* = 1664; 5.5%) and Germany (*n* = 149; 0.5%). Consumers submitted 23,587 reports (77.4%), healthcare professionals submitted 3089 (10.1%), and reporter type was unspecified in 3778 (12.4%). Female sex was reported in 20,166 reports (66.2%) and male sex in 3033 (10.0%), and sex was unspecified in 7255 (23.8%). Age was available for 15,995 reports (52.5%); the median age was 48 years.

Among the 16,684 primary-suspect reports, 5294 (31.7%) were classified as serious. Seriousness outcomes were not mutually exclusive, and 508 serious reports contained more than one outcome category. Using all 16,684 primary-suspect reports as the denominator, the “other serious outcome” category was recorded in 3624 reports (21.7%), hospitalisation in 1657 (9.9%), disability in 229 (1.4%), life-threatening events in 203 (1.2%), death in 130 (0.8%), congenital anomaly in 31 (0.2%), and required intervention in 29 (0.2%). Category-specific counts should not be summed because outcomes could overlap within a report.

### 2.2. Temporal Distribution of Adverse Event Reports

**Figure 1:**
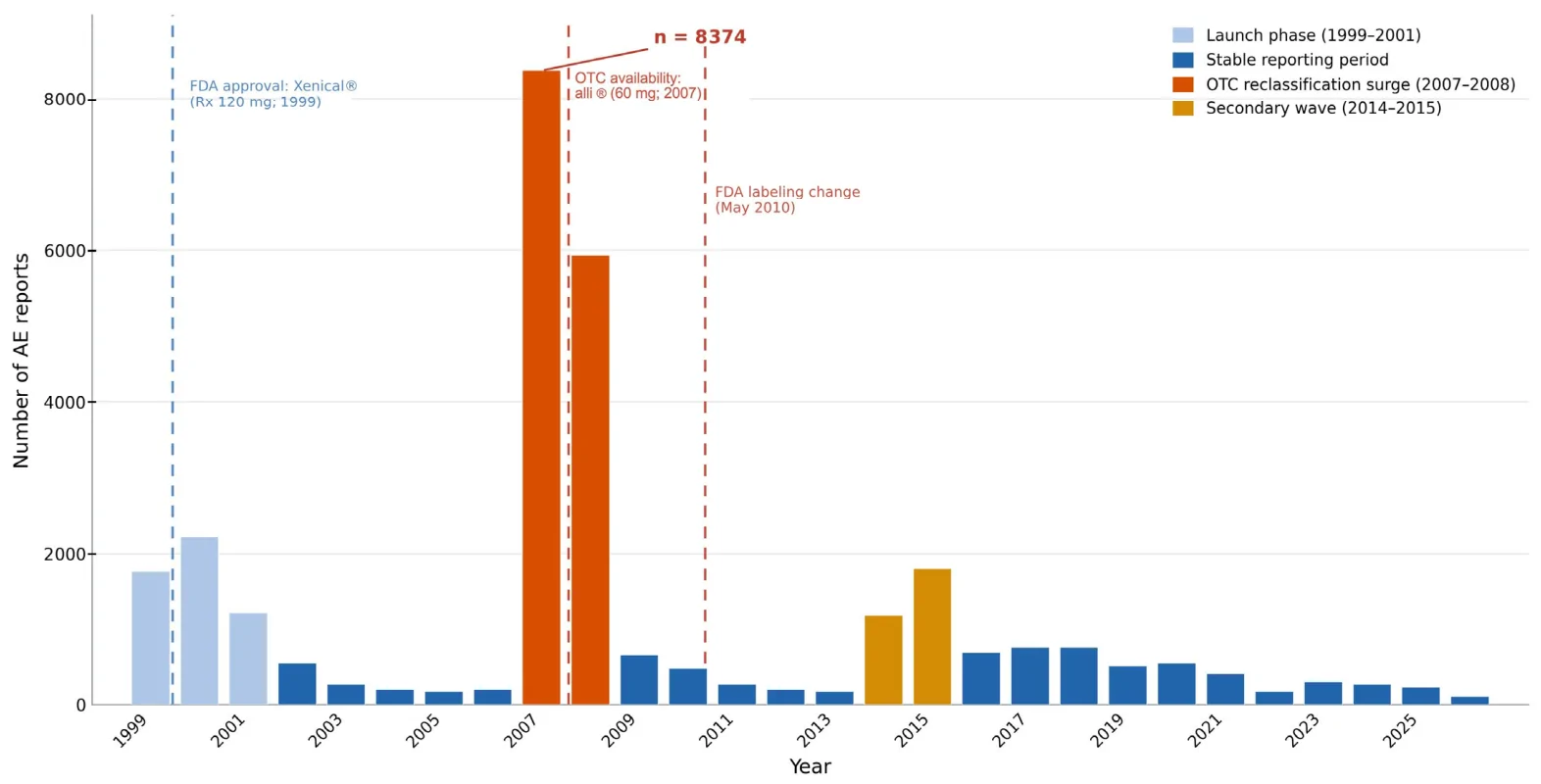
Annual distribution of orlistat-associated adverse event reports in the FDA Adverse Event Reporting System (FAERS), 1999–2026. Bars represent the total number of deduplicated reports per calendar year. Three regulatory milestones are indicated by dashed vertical lines: the initial U.S. approval of Xenical^®^ 120 mg orlistat in 1999 [11], the OTC availability of orlistat 60 mg (alli^®^; 2007) [16], and the FDA labelling change regarding orlistat-associated severe liver injury (May 2010) [17]. The 2007 reporting peak (*n* = 8374) corresponds to an approximately 41-fold increase compared with 2006.

The annual distribution of orlistat-associated adverse event reports varied substantially over the study period (Figure 1). During 1999–2001, 5228 reports were recorded, with the highest early annual counts observed in 1999 (*n* = 1787) and 2000 (*n* = 2230) following the initial U.S. approval of Xenical^®^ 120 mg orlistat in 1999 [11]. Annual reporting declined thereafter, reaching 166 reports in 2005 and remaining low immediately before OTC availability, with 204 reports recorded in 2006. After orlistat became available as the OTC 60 mg formulation alli^®^ in 2007 [16], reporting increased sharply to 8374 reports, corresponding to an approximately 41-fold increase from the previous year. Reports remained elevated in 2008 (*n* = 5975) but decreased rapidly in subsequent years. A secondary, lower-magnitude increase was observed in 2014–2015 (*n* = 2988 combined). From 2016 onwards, reporting stabilised at approximately 80–100 reports per quarter.

### 2.3. Disproportionality Analysis Results

**Figure 2:**
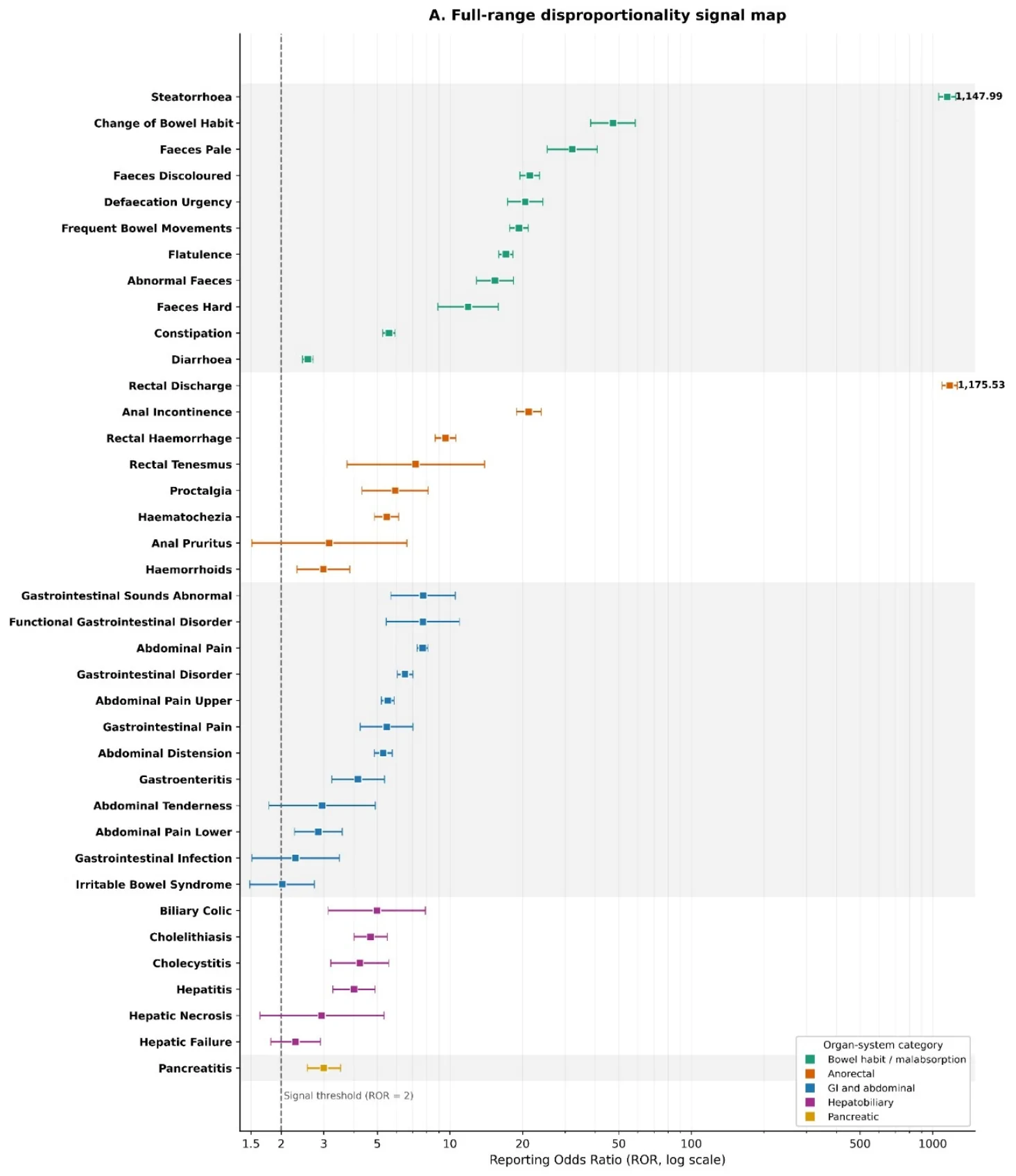

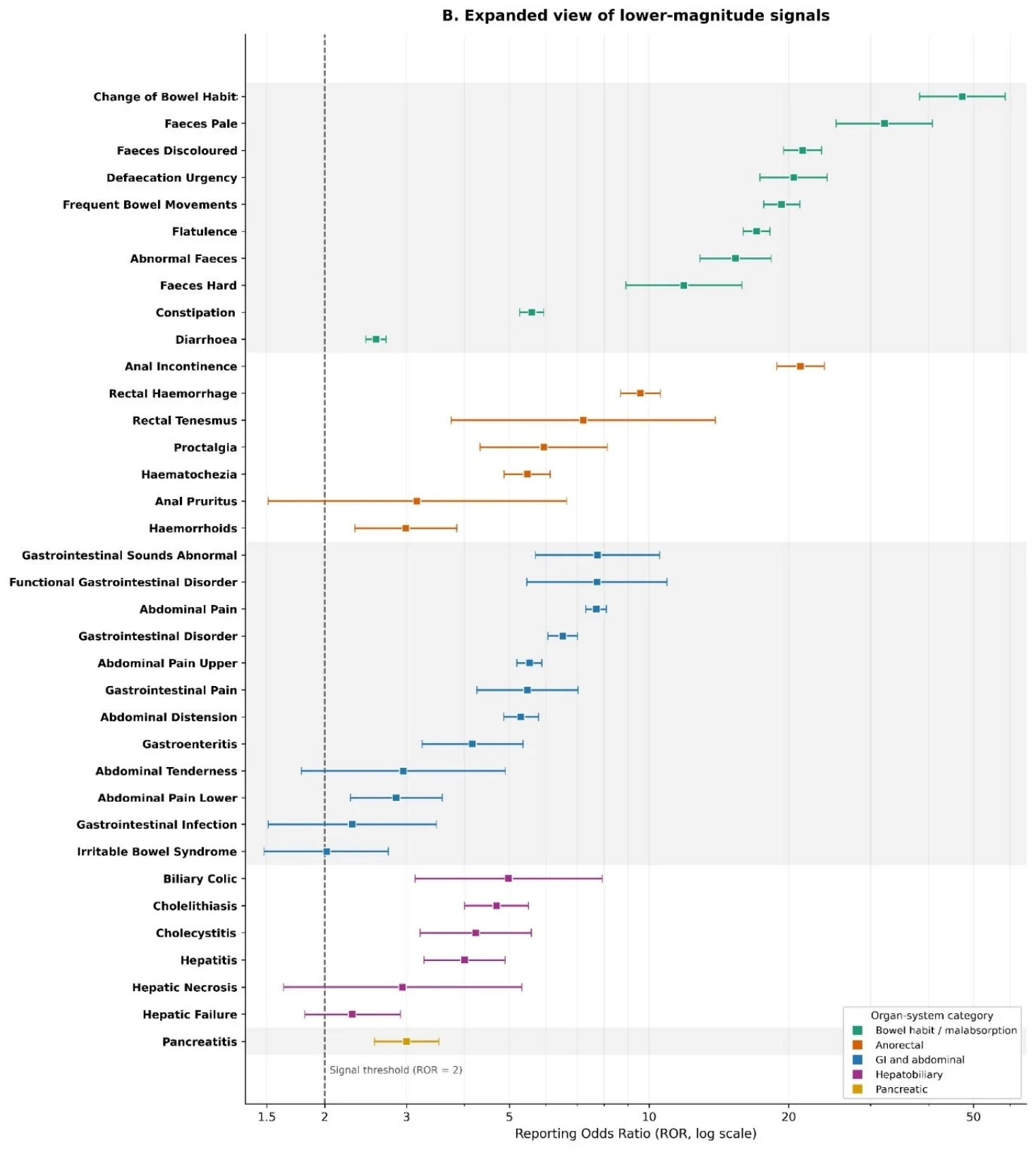
Disproportionality signal map of the 38 signal-positive MedDRA preferred terms associated with orlistat. Squares indicate ROR estimates, and horizontal error bars indicate 95% confidence intervals. Terms are colour-coded by clinical category, and the *x*-axis is presented on a logarithmic scale. The dashed vertical line denotes the ROR threshold of 2. All displayed PTs met the prespecified signal criteria of ROR > 2, lower bound of the 95% CI > 1, and *n* ≥ 3. (**A**) Full-range view, including the extreme signals for rectal discharge and steatorrhoea. (**B**) Expanded view of the remaining lower-magnitude signals.

Of the 65 prespecified MedDRA PTs, 38 met the pharmacovigilance signal threshold (ROR > 2, lower bound of the 95% CI > 1, and *n* ≥ 3). All 38 remained statistically significant after Benjamini–Hochberg false discovery rate correction (q < 0.05). Fourteen additional PTs had Benjamini–Hochberg-adjusted q values below 0.05 but were not classified as pharmacovigilance signals because they did not meet the complete prespecified signal criteria. Signal-positive PTs are presented in Table 1 and Figure 2. Complete disproportionality results for all 65 PTs, including the 27 non-signal terms, are provided in Supplementary Table S1; unadjusted *p* values and Benjamini–Hochberg-adjusted q values are provided in Supplementary Table S2.

Rectal discharge (ROR 1175.53; 95% CI 1093.44–1263.78; *n* = 1640) and steatorrhoea (ROR 1147.99; 95% CI 1058.12–1245.50; *n* = 1278) had the largest estimates. Other high-magnitude signals included change in bowel habit, faeces pale, faeces discoloured, anal incontinence, defaecation urgency, and frequent bowel movements. This overall reporting pattern is broadly consistent with the known gastrointestinal effects of orlistat and prior FAERS evidence [12].

Among hepatobiliary events, biliary colic (ROR 4.98; *n* = 18), cholelithiasis (ROR 4.69; *n* = 156), cholecystitis (ROR 4.23; *n* = 51), and hepatitis (ROR 4.00; *n* = 96) showed disproportionate reporting. Pancreatitis (ROR 3.00; *n* = 155) was the only pancreatic PT meeting the signal threshold. PRR estimates were directionally consistent with the corresponding RORs. Figure 2 displays all 38 signal-positive PTs, while the complete 2 × 2 data and disproportionality statistics for all 65 screened PTs are provided in Supplementary Table S1.

### 2.4. Cross-Referencing with Regulatory Labels

**Figure 3:**
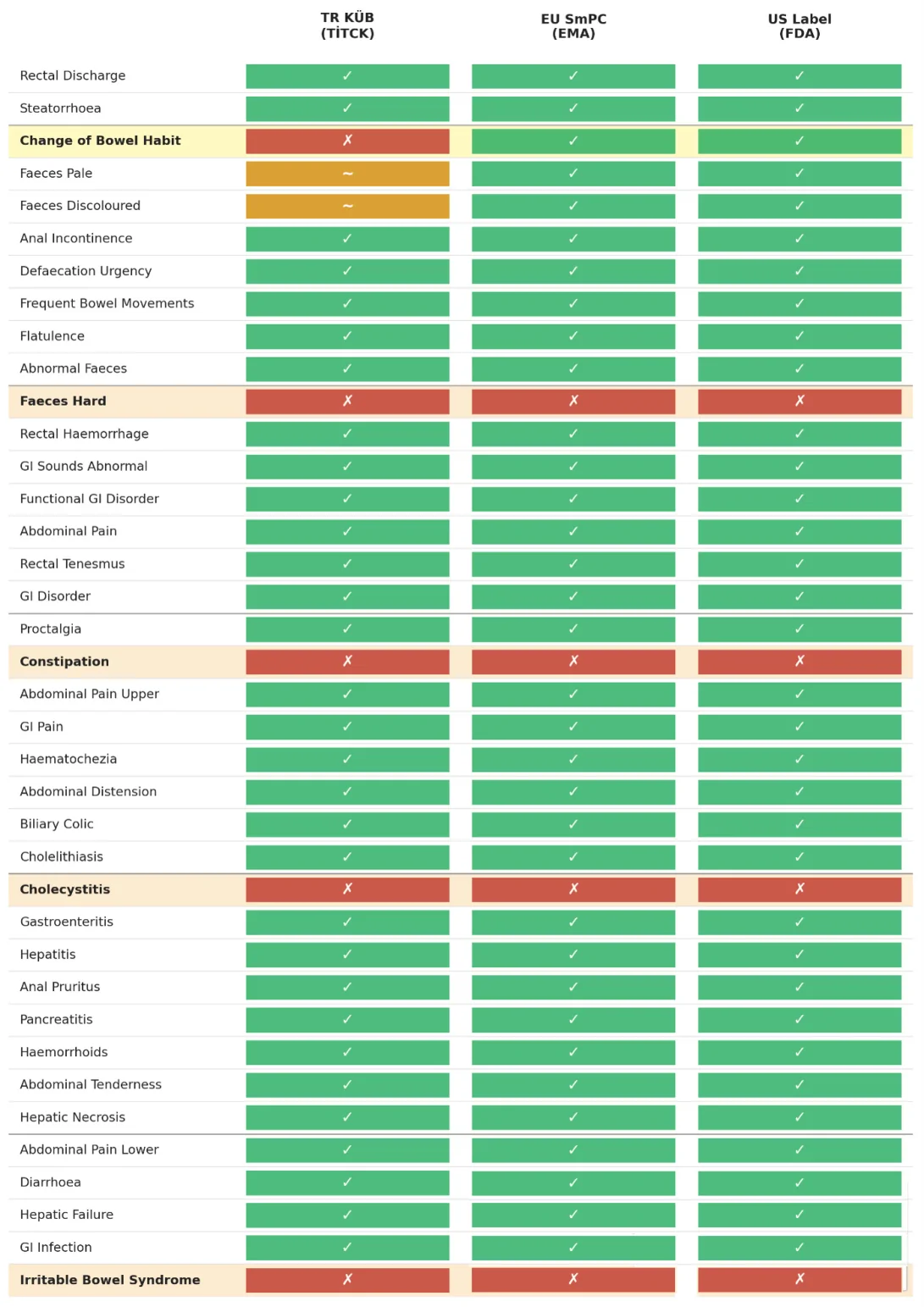
Cross-jurisdictional label concordance map for signal-positive orlistat adverse events. Each row represents a MedDRA PT meeting the signal threshold (ROR > 2, lower bound of the 95% CI > 1, and *n* ≥ 3). Columns represent Türkiye (KÜB; TİTCK), the European Union (SmPC; EMA), and the United States (Prescribing Information; FDA). Green (✓) indicates that the event was listed, red (✗) that it was absent, and yellow (~) that it was not listed verbatim but was covered by broader equivalent wording. Orange row shading identifies PTs absent from all three labels and requiring independent confirmation before regulatory interpretation; yellow row shading identifies jurisdiction-specific label discordance.

Systematic cross-referencing of the 38 signal-positive PTs with orlistat prescribing information from Türkiye, the European Union, and the United States revealed three main categories of regulatory concordance: PTs listed or clinically covered in all three jurisdictions, PTs absent from the Turkish KÜB but listed in the European and US prescribing information, and signal-positive PTs absent from all three prescribing-information documents (Figure 3, Table 2).

Four signal-positive PTs were absent from all three prescribing-information documents and were therefore classified as unlabelled signal-positive PTs: constipation (ROR 5.59; 95% CI 5.26–5.93; PRR 5.27; *n* = 1133), faeces hard (ROR 11.88; 95% CI 8.90–15.84; PRR 11.85; *n* = 47), cholecystitis (ROR 4.23; 95% CI 3.21–5.57; PRR 4.22; *n* = 51), and irritable bowel syndrome (ROR 2.02; 95% CI 1.48–2.74; PRR 2.01; *n* = 41). These PTs met the prespecified disproportionality signal criteria but were not listed in any of the three evaluated prescribing-information documents.

Change in bowel habit represented a jurisdictionally discordant signal. Although it was one of the strongest signals in the dataset (ROR 47.32; 95% CI 38.27–58.51; *n* = 90), this PT was absent from the Turkish prescribing information but listed in both the European SmPC and the US Prescribing Information.

## 3. Discussion

Using FAERS data from 2004 Q1 through 2026 Q1, we systematically evaluated gastrointestinal, hepatobiliary, pancreatic, and anorectal adverse-event reports associated with orlistat. Of the 65 MedDRA preferred terms evaluated, 38 met the prespecified disproportionality signal criteria. A substantial proportion of these signals was consistent with the expected pharmacological effects of intestinal lipase inhibition and fat malabsorption [1,3]. Alongside these expected fat-malabsorption-related events, signals were also detected for constipation, hard stools, cholecystitis, irritable bowel syndrome, pancreatitis, and anorectal complaints, some of which are already represented in current prescribing information and others of which warrant further pharmacovigilance evaluation. As with all spontaneous-reporting analyses, these findings represent disproportionate reporting rather than incidence, absolute clinical risk, or proof of causality [13,14,15].

Re-evaluating orlistat in the current clinical context has become particularly relevant as obesity pharmacotherapy increasingly intersects with liver disease management, particularly NAFLD/MASLD. Glucagon-like peptide-1 (GLP-1) receptor agonists and other incretin-based agents produce greater weight loss than orlistat in the treatment of obesity [4]. However, the FDA approval of semaglutide for adults with MASH and moderate-to-advanced liver fibrosis marks a new clinical era in which weight-management drugs are also being evaluated in terms of liver disease modification [5]. Although orlistat produces more modest weight loss, it has a distinct safety profile because of its low systemic exposure and primary site of action within the gastrointestinal lumen [1,3]. Potential benefits on hepatic steatosis, liver enzymes, and certain metabolic parameters have also been reported in patients with NAFLD/MASLD [6,7,8]. Therefore, detailed evaluation of real-world safety data on orlistat remains relevant when considering its possible role in this clinical context.

The temporal and demographic distribution of reports provides additional context for interpreting the FAERS findings. The most prominent increase in reporting occurred after the introduction of over-the-counter low-dose orlistat in 2007, when report counts increased from 204 in 2006 to 8374 in 2007 before declining markedly in subsequent years [16]. This pattern is more consistent with changes in product access, consumer awareness, and reporting behaviour than with a sustained increase in adverse-event incidence [15,16,18]. Given that FAERS is a U.S. spontaneous-reporting system and reporting from other countries is not mandatory, the predominance of reports originating from the United States was expected. The high proportion of consumer reports may nevertheless reflect, at least in part, the influence of OTC availability and U.S. reporting practices. Most reports involved female patients (66.2%), and the median age among reports with available age information was 48 years. Notably, 31.7% of primary-suspect reports met FAERS seriousness criteria, indicating that the reporting profile was not limited to mild gastrointestinal tolerability complaints; however, seriousness classification in FAERS does not establish attribution of the reported outcome to orlistat [15].

The strongest signals were detected for events directly reflecting fat malabsorption, such as rectal discharge and steatorrhoea. Orlistat inhibits gastric and pancreatic lipases, thereby reducing the hydrolysis of dietary triglycerides into absorbable free fatty acids and monoglycerides; consequently, unabsorbed fat remains within the intestinal lumen and is excreted in the stool [1,3]. This mechanism explains typical orlistat-associated gastrointestinal adverse events such as oily spotting, flatus with discharge, faecal urgency, fatty/oily stool, increased defaecation frequency, and faecal incontinence [3,11]. The American Gastroenterological Association guideline also states that treatment discontinuation due to adverse events is more frequent among patients receiving orlistat than among controls and is predominantly related to gastrointestinal tolerability problems [9]. Accordingly, the signals for rectal discharge and steatorrhoea are consistent with the established pharmacological effects of orlistat. Signals such as defaecation urgency, frequent bowel movements, flatulence, faecal discolouration, and change in bowel habit further demonstrate that the gastrointestinal reporting profile extends beyond fatty stool alone and encompasses a broader range of potentially relevant tolerability concerns [9,11].

The preferred term “change of bowel habit” reflects a general alteration in stool consistency, defaecation frequency, and bowel pattern rather than a single specific adverse event. Its inclusion in the European and US prescribing information, but not in the Turkish KÜB, represents a noteworthy jurisdiction-specific difference in terminology and coverage [11,19,20]. Irritable bowel syndrome, in contrast, was not listed in any of the three prescribing-information documents and therefore represents an unlabelled signal of particular interest [11,19,20]. Changes in intraluminal fat content, gas production, stool consistency, and bowel frequency provide a biologically plausible context for the observed reporting pattern [3,11]. However, pre-existing functional bowel symptoms, dietary changes, concomitant medications, and other patient-related factors may also have contributed. The observed association therefore merits further evaluation in data sources capable of addressing these alternative explanations.

The constipation signal is one of the most notable findings of this study because it appears paradoxical in relation to the expected pharmacological effect of orlistat. Orlistat increases faecal fat content and is commonly associated with increased defaecation frequency and looser or oily stools [1,3]. Accordingly, orlistat has been explored as a potential treatment for constipation in selected clinical contexts. In a three-case series reported by Guarino, marked improvement in bowel function was observed after orlistat use in obese patients with opioid-associated refractory constipation [21]. Iqbal et al. reported that orlistat may improve bowel function in selected patients with severe slow-transit chronic idiopathic constipation who were being evaluated for surgical intervention [22]. In addition, a randomised controlled study in clozapine-induced constipation and a more recent randomised clinical trial in patients with obesity, diabetes, and constipation suggested potentially beneficial effects of orlistat on constipation symptoms [23,24].

Against this clinical background, the constipation signal based on 1133 reports provides a notable contrast to the predominantly fat-malabsorption-related gastrointestinal profile of orlistat. The accompanying hard-stool signal further indicates that a subset of reports reflects a bowel pattern distinct from the classic fat-malabsorption profile. Packard et al. reported constipation, polyuria, polydipsia, and oedema after initiation of orlistat, with resolution after discontinuation and recurrence after rechallenge; the association was classified as “possible” according to the Naranjo probability scale [25]. In the FAERS-based analysis by Li et al., 1918 constipation reports were identified for orlistat, positive signals were obtained across four signal-detection algorithms, and the median time to onset was three days [10]. These observations provide additional context for considering constipation as a pharmacovigilance finding worthy of further investigation.

Several alternative explanations should nevertheless be considered. Orlistat is used alongside a reduced-calorie diet with controlled fat intake [9,11]. During weight-management treatment, changes in dietary fat, fibre and fluid intake, meal composition, and physical activity may alter bowel habits independently of the pharmacological action of orlistat [26,27]. Concomitant medications with constipating effects may also have contributed to the observed reports [28]. These factors may be particularly relevant in populations undergoing obesity treatment. In a cohort of adults with class II or III obesity, constipation was common and was significantly associated with polypharmacy [29]. Gastrointestinal symptoms, including functional bowel symptoms, may also coexist in individuals participating in weight-management programmes and may be associated with dietary and physical-activity patterns [27]. However, a meta-analysis found no significant association between higher body mass index and constipation or hard stools, suggesting that obesity alone is unlikely to provide a sufficient explanation for the observed signal [30]. An additional source of potential confounding is the treatment indication itself. Because orlistat has been investigated as a treatment for constipation in selected clinical settings [21,22,23,24], some reports coded with constipation as an adverse event could theoretically reflect persistence or non-resolution of a pre-existing constipation indication rather than a newly occurring event. Concomitant medications and comorbidities may likewise have contributed to individual reports. Report-level descriptive analyses of treatment indication, concomitant medications, and relevant comorbidities were not performed in the present study; therefore, the extent to which these factors contributed to the observed constipation signal could not be quantified. These considerations further support cautious interpretation of the signal and evaluation in data sources that allow more detailed assessment of indication, medication exposure, comorbidity, and temporal relationships.

The signals for rectal haemorrhage, haematochezia, haemorrhoids, proctalgia, anal pruritus, and rectal tenesmus broaden the gastrointestinal safety profile observed in FAERS beyond the classic manifestations of fatty stools and diarrhoea. Changes in defaecation frequency, stool consistency, faecal urgency, and local irritation associated with fat malabsorption provide a biologically plausible clinical context for these reports [3,11]. The FDA prescribing information also notes that lower gastrointestinal bleeding has been reported in patients receiving orlistat [11]. Taken together, these findings identify anorectal tolerability as an aspect of the orlistat reporting profile that warrants further focused clinical investigation, while haemorrhoidal disease, local irritation, altered bowel patterns, and other patient-related factors may also have contributed to the reported events [15].

The upper gastrointestinal reporting pattern was less pronounced than the lower gastrointestinal reporting pattern. Although the classic safety profile of orlistat is mainly characterised by complaints related to bowel pattern and fat malabsorption, clinical and post-marketing studies have also reported a broader range of gastrointestinal adverse events, including abdominal pain, dyspepsia, bloating, and nausea/vomiting [31,32]. In our study, upper gastrointestinal preferred terms such as dyspepsia, eructation, gastritis, gastric ulcer, regurgitation, gastro-oesophageal reflux disease, and vomiting did not meet the prespecified pharmacovigilance signal criteria. Although some of these PTs had Benjamini–Hochberg-adjusted q values below 0.05, they did not satisfy the complete prespecified criteria for pharmacovigilance signal classification. Upper gastrointestinal complaints are non-specific and may be influenced by obesity-associated reflux tendency, dietary and physical-activity patterns, and background reporting behaviour in spontaneous-reporting systems [13,15,27,30]. Accordingly, these findings complement the overall gastrointestinal safety assessment but remain non-signal findings under the prespecified pharmacovigilance definition [14,15].

Positive disproportionality signals were also detected for hepatobiliary and pancreatic events, including cholelithiasis, cholecystitis, biliary colic, pancreatitis, hepatitis, hepatic failure, and hepatic necrosis. Although reported less frequently than the dominant gastrointestinal events, these findings extend the observed reporting profile to clinically important hepatobiliary and pancreatic outcomes. They are generally consistent with the FAERS analysis by Zhu et al., in which gastrointestinal and hepatobiliary events were identified as prominent categories among orlistat-associated reports [12].

Interpretation of the hepatic signals requires consideration of the broader clinical context of orlistat use. The FDA prescribing information contains warnings regarding rare but important events such as severe liver injury, hepatocellular necrosis, and acute hepatic failure [11]. Sall et al. also described a case of fulminant hepatic failure requiring liver transplantation after two months of OTC orlistat use [33]. In contrast, some studies have suggested potentially beneficial effects of orlistat on hepatic steatosis, liver enzymes, and certain metabolic parameters in patients with NAFLD/MASLD. In a randomised placebo-controlled trial, Zelber-Sagi et al. observed improvements in serum alanine aminotransferase levels and ultrasonographic steatosis in patients with NAFLD [34]. More recent systematic reviews and meta-analyses have provided additional evidence, including reductions in liver fat content, ALT, AST, total cholesterol, and LDL-C levels [6], together with potential improvements in AST, ALT, and other metabolic parameters in patients with MASLD, although these findings should be interpreted in light of study heterogeneity [7]. A GRADE-assessed meta-analysis by Karimi et al. likewise found favourable effects on ALT, AST, GGT, and insulin-resistance parameters while noting the need for larger studies to evaluate long-term safety [8]. Taken together, this literature indicates that hepatic adverse-event signals should be interpreted within a balanced clinical context that incorporates both recognised safety concerns and reported metabolic or hepatic benefits in patients with NAFLD/MASLD.

The hepatobiliary signals detected in this study may also be substantially influenced by confounding by indication and underlying disease. Orlistat is prescribed for obesity, and patients receiving it may also have type 2 diabetes, metabolic syndrome, NAFLD/MASLD, or pre-existing gallstone disease. These conditions are associated with elevated baseline risks of hepatic or biliary outcomes. Obesity and rapid weight loss are established risk factors for gallstone formation [35]. Longitudinal evidence has also linked metabolic syndrome to incident gallstone disease, although the strength of this association may vary by sex [36]. NAFLD and gallstone disease have shown a bidirectional epidemiological association, with each condition associated with the subsequent development of the other [37]. Type 2 diabetes has likewise been associated with symptomatic gallstone disease and an increased risk of severe liver disease [38,39]. Moreover, pre-existing gallstones may lead to biliary colic and complications such as acute cholecystitis, biliary obstruction, cholangitis, or pancreatitis [35]. Because FAERS reports often contain incomplete information on treatment indication, baseline liver and gallbladder status, comorbidity severity, the magnitude and rate of weight loss, and the temporal sequence of these factors relative to the reported event, their individual contributions cannot be separated reliably in the present analysis [13,15,40]. The observed hepatobiliary disproportionality should therefore be viewed within this elevated baseline-risk context rather than attributed to a single explanatory factor.

The gallbladder-related signals are particularly noteworthy given the multiple clinical and physiological factors relevant to gallstone disease. Obesity, rapid weight loss, and impaired gallbladder emptying are established risk factors for gallstone formation [35]. Previous physiological studies have suggested that lipase inhibition may reduce postprandial cholecystokinin release and gallbladder emptying [41,42]. In the randomised study by Mathus-Vliegen et al., orlistat at doses of 60 mg and 120 mg was associated with 15% and 53% reductions in gallbladder emptying, respectively, together with a reduced cholecystokinin response [41]. These physiological observations provide a biologically plausible context for considering altered gallbladder motility as one possible contributor to the reporting pattern, alongside obesity, rapid weight loss, pre-existing gallstone disease, metabolic comorbidities, and concomitant medications. Cholecystitis is particularly notable because it met the prespecified signal criteria but was not listed as a separate term in the US, European, or Turkish prescribing information [11,19,20]. This combination of disproportionality, a biologically plausible physiological context, and absence as a discrete labelled term makes cholecystitis a focused candidate for further clinical and epidemiological evaluation.

The pancreatitis signal is also clinically relevant. Pancreatitis is described in the FDA prescribing information as a post-marketing event, and published case reports have documented acute pancreatitis following orlistat exposure [11,43,44,45]. In the present analysis, pancreatitis was the only pancreatic PT that met the prespecified signal criteria. At the same time, obesity, gallstone disease, metabolic risk factors, and concomitant medications may independently contribute to pancreatitis in the population receiving orlistat [15,35]. The observed signal therefore identifies pancreatitis as a priority for further focused clinical and epidemiological investigation, while the relative contributions of treatment and underlying patient risk cannot be resolved from FAERS data alone.

Comparison of the detected signals with prescribing information from Türkiye, the European Union, and the United States provides an additional regulatory context for the findings. Constipation, faeces hard, cholecystitis, and irritable bowel syndrome were signal-positive PTs that were not included in the prescribing information of any of the three jurisdictions [11,19,20]. In contrast, change in bowel habit is included in the European and US prescribing information but not in the Turkish KÜB, indicating a jurisdiction-specific difference in terminology and coverage. These findings identify areas in which the FAERS reporting profile and current regulatory terminology do not fully overlap. Such discordance is informative for prioritising future clinical and pharmacovigilance evaluation, but spontaneous-reporting signals alone are insufficient to establish a need for regulatory label revision [13,14,15].

The difference between the MedDRA coding level and the wording used in prescribing information should also be considered. MedDRA enables adverse events to be coded using standardised preferred terms [46,47], whereas prescribing information may describe the same clinical concepts using broader adverse-event terminology. Consequently, exact PT-level concordance cannot always be expected. Several screened PTs were not listed verbatim in the prescribing-information documents examined, although the underlying clinical concepts may have been represented by broader gastrointestinal or hepatic wording [11,19,20]. Label comparisons should therefore consider clinical equivalence and terminology granularity rather than relying solely on exact lexical correspondence [46,47].

A similar FAERS-based evaluation of the safety profile of orlistat was published by Zhu et al. Their study analysed FAERS data from 2004 Q1 to 2023 Q1, evaluated reports in which orlistat was designated as the primary-suspect drug, and detected PT-level signals across different system organ classes [12]. The present study differs from that analysis in several respects. First, the study period was extended to 2026 Q1, providing a more up-to-date FAERS data range. Second, rather than conducting a general safety assessment across all system organ classes, the present study focused specifically on gastrointestinal, hepatobiliary, pancreatic, and anorectal adverse events. Third, detected signals were compared with prescribing information from Türkiye, the European Union, and the United States, enabling identification of jurisdiction-specific differences in terminology and coverage. Finally, constipation, hepatobiliary signals, pancreatitis, upper gastrointestinal symptoms, and anorectal events were interpreted in the context of relevant clinical and mechanistic literature rather than presented solely as numerical pharmacovigilance findings.

### Strengths and Limitations

The strengths of this study include its broad time span, use of up-to-date FAERS data, deduplication of a large dataset, prespecification of 65 MedDRA PTs, use of complementary ROR and PRR measures, application of Benjamini–Hochberg false discovery rate correction, and structured comparison with prescribing information from three regulatory jurisdictions. This structured comparison enabled the identification of unlabelled and jurisdictionally discordant signal-positive PTs and the characterisation of terminological differences among prescribing-information documents. Interpretation of constipation, gallbladder and pancreatic events, anorectal events, and hepatic signals in relation to the relevant clinical literature provides additional clinical context for the pharmacovigilance findings.

Several limitations inherent in spontaneous-reporting data should be considered. FAERS is subject to underreporting and selective reporting, and the likelihood of report submission may vary according to event severity, reporter type, time since market introduction, media attention, and changes in product availability [13,15,48]. Regulatory safety communications and labelling changes may additionally stimulate reporting for particular products or drug–event pairs, although this effect is not uniform across FAERS [18]. Consequently, temporal changes in report counts cannot be interpreted as corresponding changes in adverse-event incidence. Report quality and completeness are also variable, and submitted information is not necessarily independently verified. Important clinical details, including treatment indication, dose, treatment duration, time to onset, dechallenge or rechallenge, laboratory findings, diagnostic confirmation, concomitant medications, and comorbidities, may be missing or inconsistently reported [15,40]. These limitations precluded reliable assessment of dose–response relationships, temporal relationships, causality, and the clinical validity of individual reports.

Because FAERS does not provide a reliable denominator for the number of exposed patients, true incidence and absolute risk cannot be calculated; RORs and PRRs quantify disproportionate reporting rather than clinical risk [13,14,15]. Residual duplicate reports may remain despite the deduplication procedure, and the recording of multiple suspected or concomitant drugs and multiple adverse events within the same report may further complicate drug–event attribution [13,15,40]. Although FAERS may contain information on treatment indication, concomitant medications, and comorbidities in individual reports, these variables are incompletely and inconsistently recorded and were not evaluated through report-level descriptive confounding analyses in the present study. Consequently, residual confounding by concomitant medications, comorbidities, disease severity, treatment indication, dietary or lifestyle factors, and other unmeasured characteristics remains an important limitation. This limitation is particularly relevant to the constipation signal because constipation may itself have been a treatment indication in some reports, and persistence of the underlying condition cannot be reliably distinguished from a newly reported adverse event without detailed report-level clinical review.

Restriction to primary-suspect reports may have improved the specificity of drug–event attribution but may also have reduced sensitivity by excluding reports in which orlistat was assigned another suspect role; signal stability across broader suspect-role definitions was not assessed [13,15,40]. The use of all other drugs as the reference group may also have introduced indication-related and reporting-related bias. An active-comparator sensitivity analysis using other anti-obesity medications was not performed because available anti-obesity medications comprise a clinically heterogeneous group with differing mechanisms, treatment contexts, market periods, and adverse-event profiles [4].

Era-, reporter-type-, country-, age-, and sex-stratified disproportionality analyses were not performed. The corresponding strata were either incomplete or markedly imbalanced, and reliable stratified analyses would also have required equivalent classification of the complete comparator database. Consequently, the pooled estimates may have been influenced by changes in product availability and reporting intensity, US-specific OTC access, consumer reporting practices, and demographic or geographic reporting-source imbalance. In particular, the predominance of US and consumer reports should not be interpreted as evidence of geographic or reporter-specific differences in clinical risk.

These limitations are particularly relevant to hepatobiliary outcomes because patients receiving orlistat for obesity may also have metabolic syndrome, type 2 diabetes, NAFLD/MASLD, or pre-existing gallstone disease, all of which may increase baseline hepatic or biliary risk [35,36,37,38,39]. FAERS cannot reliably establish the temporal relationship of these conditions to treatment initiation and the reported event, characterise their severity, or consistently capture baseline liver or gallbladder status or the magnitude and rate of weight loss. Accordingly, the detected signals should be interpreted as pharmacovigilance findings that identify patterns of disproportionate reporting and priorities for further investigation. Confirmation of their clinical implications will require studies with validated outcomes, reliable exposure denominators, and adequate control of confounding.

## 4. Materials and Methods

### 4.1. Study Design and Data Source

This retrospective pharmacovigilance study was conducted using the publicly available FAERS database. FAERS is a voluntary spontaneous reporting system that contains individual case safety reports submitted to the FDA by healthcare professionals, consumers, and pharmaceutical manufacturers, and is widely used as a publicly accessible source for post-marketing adverse drug event surveillance [13,15].

All quarterly FAERS data files from 2004 Q1 through 2026 Q1 were downloaded from the FDA open-data portal and integrated into a unified relational database (PostgreSQL v15) for preprocessing and analysis [49]. During the study period, the FDA announced the launch of the Adverse Event Monitoring System (AEMS), a unified platform intended to modernise adverse event reporting across FDA-regulated products; however, the present analysis was conducted using the publicly available FAERS quarterly data files through 2026 Q1 [50]. FAERS is structured around seven relational files: demographic information (DEMO), drug details (DRUG), adverse reaction terms (REAC), patient outcomes (OUTC), indication data (INDI), report sources (RPSR), and medication dates (THER), which were linked using the report identifiers provided in the quarterly FAERS files [13,49]. This study was conducted exclusively on publicly available, de-identified surveillance data and did not require ethical approval or informed patient consent.

### 4.2. Data Processing and Deduplication

Prior to analysis, duplicate reports were removed using the FAERS demographic file (DEMO). Records were sorted by CASEID in ascending order, FDA_DT in descending order, and PRIMARYID in descending order. Reports representing multiple versions of the same case were not treated as independent records. For cases with partially discordant report versions, the most recent follow-up version was retained as the representative record based on FDA_DT; when FDA_DT values were identical, the record with the highest PRIMARYID was selected. Thus, each CASEID contributed only one retained record to the analytical dataset. This procedure was consistent with previously described FAERS deduplication approaches and FDA quarterly data-file handling practices [13,49].

Drug-name normalisation used the “Suspect Product Names” and “Suspect Product Active Ingredients” fields in the DRUG file. Entries were trimmed and matched case-insensitively. ORLISTAT was used as the exact active-ingredient identifier, while ALLI and XENICAL were the only recognised brand names observed in the dataset. All included reports explicitly contained ORLISTAT in the active-ingredient field. No additional spelling variants were identified. Records without an exact ORLISTAT active-ingredient match were excluded; missing or alternative product-name entries were retained only when ORLISTAT was explicitly documented as an active ingredient. Multiple qualifying drug entries within the same deduplicated CASEID were counted once. The complete drug-name normalisation rules, recognised brand names, and exclusion criteria used to define the orlistat-associated population are provided in Supplementary Table S3.

Adverse reaction terms in the REAC file were mapped to Medical Dictionary for Regulatory Activities version 27.0 (MedDRA v27.0) preferred terms (PTs) and system organ classes (SOCs) to ensure standardised event classification [46].

Missing age and sex values were not imputed. Age summaries were calculated using available-case denominators, and sex was classified as female, male, or not specified. Because age and sex were used only for descriptive characterisation and were not included in the drug–event case definition or the 2 × 2 contingency tables, eligible reports were retained irrespective of missing demographic information. A complete-case restriction was not applied to avoid excluding otherwise eligible reports solely because of missing demographic information. Seriousness status and individual outcomes were obtained from the corresponding FAERS fields. Seriousness outcomes were treated as non-mutually exclusive because more than one outcome category could be recorded for a single report. Outcome-specific percentages used all 16,684 primary-suspect reports as the denominator.

### 4.3. Case Identification and Adverse Event Selection

Reports involving orlistat were identified by searching standardised drug name fields within the DRUG file. All deduplicated orlistat-associated reports were used for descriptive temporal and demographic analyses. For disproportionality analyses, only reports in which orlistat was designated as the primary suspect drug (role code = ‘PS’) were retained to reduce the influence of concomitant or interacting medications and to increase the specificity of drug–event attribution [13,40].

Candidate adverse-event concepts were identified based on three sources: (i) the pharmacological mechanism of orlistat and its expected fat-malabsorption-related effects; (ii) adverse reactions and post-marketing events described in the Turkish, European Union, and United States prescribing information; and (iii) relevant clinical and pharmacovigilance literature [1,3,11,12,19,20]. These concepts were mapped to MedDRA version 27.0 preferred terms and grouped into four predefined clinical domains: gastrointestinal and abdominal events, bowel-habit and malabsorption-related events, anorectal events, and hepatobiliary or pancreatic events [46,47]. The candidate clinical domains were defined a priori, and the final list of 65 MedDRA preferred terms was completed before calculation and interpretation of RORs, PRRs, confidence intervals, and signal status. The complete final list, including both signal-positive and non-signal terms, is provided in Supplementary Table S1.

### 4.4. Disproportionality Analysis

All non-orlistat reports were retained as the reference group. An active-comparator group comprising other anti-obesity medications was not used because available agents comprise a clinically heterogeneous group with differing mechanisms and adverse-event profiles [4].

Signal detection was performed using two established frequentist measures of disproportionality: the reporting odds ratio (ROR) and the proportional reporting ratio (PRR). For each adverse event PT, a standard 2 × 2 contingency table was constructed comparing primary-suspect orlistat reports with reports involving all other medications in the FAERS reference dataset (Table 3).

The ROR and its 95% confidence interval were calculated as follows [14]:

ROR = (a × d)/(b × c)

95% CI = exp[ln(ROR) ± 1.96 × √(1/a + 1/b + 1/c + 1/d)]

The PRR was calculated as follows [51]:

PRR = [a/(a + b)]/[c/(c + d)]

A conservative, prespecified pharmacovigilance signal definition was applied: ROR > 2.0, lower bound of the 95% CI > 1.0, and *n* ≥ 3 [14,51,52]. PRR values were reported as a complementary measure of signal consistency [51].

For each PT, a two-sided unadjusted *p* value was calculated using Fisher’s exact test applied to the corresponding 2 × 2 contingency table used for the ROR analysis. Fisher’s exact test was selected because the expected cell count was below 5 for 11 of the 65 prespecified PTs. Pearson chi-square, Yates-corrected chi-square, and Wald tests were performed as sensitivity analyses and yielded concordant significance classifications for all 65 PTs. To address multiplicity across the 65 prespecified PTs, the unadjusted *p* values were corrected using the Benjamini–Hochberg false discovery rate procedure [53]. A Benjamini–Hochberg-adjusted q value < 0.05 was considered statistically significant. FDR correction was used as a complementary robustness assessment and did not replace the primary pharmacovigilance signal definition. Signal-positive PTs are presented in Table 1; complete disproportionality results for all 65 PTs are provided in Supplementary Table S1, and unadjusted *p* values and Benjamini–Hochberg-adjusted q values are provided in Supplementary Table S2.

### 4.5. Cross-Referencing with Regulatory Prescribing Information

All signal-positive PTs were cross-referenced with three regulatory documents: (i) the Turkish Kısa Ürün Bilgisi (KÜB) for Xenical 120 mg sert kapsül, issued by the Turkish Medicines and Medical Devices Agency (TİTCK); (ii) the 2022 Xenical European Public Assessment Report Product Information, including the Summary of Product Characteristics (SmPC), published by the European Medicines Agency (EMA); and (iii) the 2022 FDA Xenical Prescribing Information (NDA 020766/S-038) [11,19,20]. All documents were accessed on 26 June 2026.

Two reviewers (H.S. and L.M.) independently assessed each signal-positive MedDRA PT in each document and classified it as: (a) listed verbatim or represented by a directly equivalent clinical term; (b) not listed verbatim but covered by broader clinically equivalent wording; or (c) absent. Broader equivalent wording was recorded separately from verbatim or directly equivalent term concordance. Disagreements were resolved through discussion and consensus; a third reviewer (B.E.A.) was available for adjudication if required. The independent reassessment did not alter the final classifications.

Based on the three-jurisdiction comparison, signal-positive PTs were grouped as: (a) listed or clinically covered in all three jurisdictions; (b) absent from the Turkish KÜB but listed in the European and US prescribing information, indicating jurisdiction-specific discordance; or (c) absent from all three documents, representing unlabelled signal-positive PTs requiring further clinical and epidemiological evaluation. The comparison was descriptive; absence of a PT from a prescribing-information document was not interpreted as sufficient evidence for regulatory label revision.

### 4.6. Temporal Analysis

The annual and quarterly distribution of orlistat-associated adverse event reports from 1999 through 2026 Q1 was characterised descriptively. Although the quarterly FAERS files analysed covered 2004 Q1–2026 Q1, retained orlistat-associated reports included Initial FDA Received Date (FDA_DT) values dating back to 1999. Temporal analyses were therefore based on FDA_DT and covered 1999 through 2026 Q1. FDA_DT was used as the temporal anchor for all time-series analyses because it reflects the date on which the report was initially received by the FDA and was more complete and consistently available in the present dataset than the event occurrence date (EVENT_DT) [49]. Three key regulatory milestones were annotated on the temporal distribution figure: the initial U.S. approval of Xenical^®^ 120 mg orlistat in 1999, the OTC availability of alli^®^ 60 mg orlistat in 2007, and the FDA labelling change regarding orlistat-associated severe liver injury in May 2010 [11,16,17].

### 4.7. Software

Data preprocessing and database management were performed using PostgreSQL v15. Statistical analyses were conducted in Python 3.12 using SciPy 1.17 for Fisher’s exact test and statsmodels 0.14 for the Benjamini–Hochberg false discovery rate procedure. Figures were generated using matplotlib and seaborn.

## 5. Conclusions

This FAERS-based study suggests that gastrointestinal events related to expected fat malabsorption remain prominent in the post-marketing reporting profile of orlistat. In addition to these expected events, signal-positive PTs included constipation, faeces hard, cholecystitis, irritable bowel syndrome, pancreatitis, and several other hepatobiliary and anorectal events. The constipation signal is notable because it contrasts with clinical reports in which orlistat has been explored as a potential treatment for constipation [21,22,23,24], while also being consistent with prior pharmacovigilance findings identifying a constipation signal for orlistat [10]. Hepatic signals should be interpreted in a balanced clinical context, considering both rare safety concerns and studies suggesting potential metabolic or hepatic benefits in NAFLD/MASLD [6,7,8]. Cross-jurisdictional comparison identified unlabelled PTs and a jurisdiction-specific labelling difference for “change in bowel habit” [11,19,20]. These label-absent and jurisdictionally discordant signals should be regarded as candidates for further clinical and epidemiological investigation rather than as sufficient evidence for regulatory label revision. Overall, independent pharmacovigilance studies, appropriately controlled observational analyses, and mechanism-focused research are needed to determine the clinical relevance of these reporting associations and to further evaluate the real-world safety profile of orlistat, including in the context of NAFLD/MASLD.

## Figures and Tables

**Table 1:** Adverse events showing a disproportionality signal for orlistat in the FAERS database (2004 Q1–2026 Q1; *n* = 16,684 primary-suspect orlistat reports; 15,680,472 comparator reports). Events are grouped by clinical category and presented with numbers of cases, reporting odds ratios, 95% confidence intervals, and proportional reporting ratios.

MedDRA Preferred Term	No. of Cases (a)	ROR (95% CI)	PRR
**Gastrointestinal and abdominal disorders**			
Abdominal Pain	1649	7.69 (7.30–8.09)	7.03
Gastrointestinal Sounds Abnormal	41	7.74 (5.69–10.53)	7.72
Functional Gastrointestinal Disorder	32	7.72 (5.45–10.93)	7.70
Gastrointestinal Disorder	741	6.51 (6.05–7.01)	6.27
Gastrointestinal Pain	61	5.46 (4.25–7.03)	5.45
Abdominal Pain Upper	1088	5.52 (5.19–5.87)	5.23
Abdominal Distension	541	5.29 (4.86–5.77)	5.15
Gastroenteritis	62	4.16 (3.24–5.35)	4.15
Abdominal Tenderness	15	2.95 (1.78–4.90)	2.95
Abdominal Pain Lower	74	2.85 (2.27–3.58)	2.84
Gastrointestinal Infection	22	2.29 (1.51–3.48)	2.29
Irritable Bowel Syndrome	41	2.02 (1.48–2.74)	2.01
**Bowel habit and malabsorption**			
Steatorrhoea	1278	1147.99 (1058.12–1245.50)	1060.13
Change in Bowel Habit	90	47.32 (38.27–58.51)	47.07
Faeces Pale	70	32.11 (25.29–40.77)	31.98
Faeces Discoloured	453	21.40 (19.47–23.51)	20.84
Defaecation Urgency	142	20.48 (17.33–24.20)	20.31
Frequent Bowel Movements	504	19.30 (17.65–21.11)	18.75
Flatulence	923	17.04 (15.93–18.21)	16.15
Abnormal Faeces	126	15.35 (12.87–18.32)	15.24
Faeces Hard	47	11.88 (8.90–15.84)	11.85
Constipation	1133	5.59 (5.26–5.93)	5.27
Diarrhoea	1558	2.58 (2.45–2.71)	2.43
**Anorectal disorders**			
Rectal Discharge	1640	1175.53 (1093.44–1263.78)	1060.08
Anal Incontinence	289	21.19 (18.84–23.83)	20.84
Rectal Haemorrhage	407	9.58 (8.68–10.58)	9.38
Rectal Tenesmus	9	7.21 (3.74–13.89)	7.20
Proctalgia	39	5.93 (4.32–8.12)	5.91
Haematochezia	302	5.46 (4.87–6.12)	5.38
Anal Pruritus	7	3.16 (1.51–6.64)	3.16
Haemorrhoids	60	2.99 (2.32–3.85)	2.98
**Hepatobiliary disorders**			
Biliary Colic	18	4.98 (3.13–7.92)	4.98
Cholelithiasis	156	4.69 (4.00–5.49)	4.65
Cholecystitis	51	4.23 (3.21–5.57)	4.22
Hepatitis	96	4.00 (3.27–4.89)	3.98
Hepatic Necrosis	11	2.94 (1.63–5.32)	2.94
Hepatic Failure	69	2.29 (1.81–2.91)	2.29
**Pancreatic disorders**			
Pancreatitis	155	3.00 (2.56–3.52)	2.98

Abbreviations: AE, adverse event; CI, confidence interval; FAERS, FDA Adverse Event Reporting System; PRR, proportional reporting ratio; ROR, reporting odds ratio. Signal threshold: ROR > 2, lower bound of the 95% CI > 1, and *n* ≥ 3.

**Table 2:** Classification of signal-positive MedDRA preferred terms according to cross-jurisdictional prescribing-information status.

Category	MedDRA Preferred Terms	Interpretation
**Listed or clinically covered in all three jurisdictions (TR, EU, US)**	Steatorrhoea, Rectal Discharge, Flatulence, Defaecation Urgency, Frequent Bowel Movements, Anal Incontinence, Faeces Pale, Faeces Discoloured, Abnormal Faeces, Diarrhoea, Abdominal Pain, Abdominal Pain Upper, Abdominal Distension, Gastrointestinal Disorder, Gastrointestinal Pain, Proctalgia, Rectal Haemorrhage, Rectal Tenesmus, Haematochezia, Haemorrhoids, Anal Pruritus, Cholelithiasis, Biliary Colic, Hepatitis, Hepatic Necrosis, Hepatic Failure, Pancreatitis, Abdominal Pain Lower, Gastroenteritis, Gastrointestinal Infection, Gastrointestinal Sounds Abnormal, Functional Gastrointestinal Disorder, Abdominal Tenderness	Listed or clinically covered in all three prescribing-information documents; consistent with existing regulatory terminology and coverage.
**Absent from Turkish KÜB; listed in EU SmPC and US label**	Change in Bowel Habit (ROR 47.32; *n* = 90)	Identifies a jurisdictional labelling difference for further comparative assessment; insufficient alone to support label revision
**Unlabelled signal-positive PTs—absent from all three labels**	Constipation (ROR 5.59; *n* = 1133), Faeces Hard (ROR 11.88; *n* = 47), Cholecystitis (ROR 4.23; *n* = 51), Irritable Bowel Syndrome (ROR 2.02; *n* = 41)	Candidates for further clinical and epidemiological investigation; insufficient alone to support regulatory label changes

EU, European Union; KÜB, Turkish prescribing information (Kısa Ürün Bilgisi); PT, preferred term; ROR, reporting odds ratio; SmPC, Summary of Product Characteristics; TR, Türkiye; US, United States.

**Table 3:** Standard 2 × 2 contingency table used for disproportionality analysis.

	Target AE Reported	Other AEs Reported
Primary-suspect orlistat reports	a	b
All other drug reports	c	d

a = primary-suspect orlistat reports containing the target AE; b = primary-suspect orlistat reports not containing the target AE; c = non-orlistat reports containing the target AE; d = non-orlistat reports not containing the target AE.

## Data Availability

The FAERS quarterly data files analysed in this study are publicly available from the U.S. Food and Drug Administration Adverse Event Monitoring System (AEMS): Latest Quarterly Data Files. The processed datasets and analysis scripts generated during the study are available from the corresponding author upon reasonable request.

## Supplementary Materials

The following supporting information can be downloaded at: https://www.mdpi.com/article/10.3390/ph19091325/s1, Table S1: Complete disproportionality analysis results for all 65 screened adverse event preferred terms. Table S2: Unadjusted *p* values and Benjamini–Hochberg false discovery rate-adjusted q values for the 65 prespecified MedDRA preferred terms. Table S3: Drug-name normalisation rules and case-definition criteria used to identify orlistat-associated reports in the FAERS database (2004 Q1–2026 Q1).

## References

[1] Heck A.M., Yanovski J.A., Calis K.A. (2000). Orlistat, a new lipase inhibitor for the management of obesity. Pharmacotherapy.

[2] World Health Organization (2024). Obesity and Overweight.

[3] Sumithran P., Proietto J. (2014). Benefit-risk assessment of orlistat in the treatment of obesity. Drug Saf..

[4] McGowan B., Ciudin A., Baker J.L., Busetto L., Dicker D., Frühbeck G., Goossens G.H., Monami M., Sbraccia P., Martinez-Tellez B. (2025). A systematic review and meta-analysis of the efficacy and safety of pharmacological treatments for obesity in adults. Nat. Med..

[5] U.S. Food and Drug Administration (2025). FDA Approves Treatment for Serious Liver Disease Known as MASH.

[6] Mahmoud A., Mohamed I., Abuelazm M., Ahmed A.A.S., Saeed A., Elshinawy M., Almaadawy O., Abdelazeem B. (2024). Efficacy of orlistat in obese patients with nonalcoholic fatty liver disease: A systematic review and meta-analysis of randomized controlled trials. Proc. Bayl. Univ. Med. Cent..

[7] Ayati A., Vahed I.E., Rahimi Y., Karbasi S., Safdari A., Razaghi M., Bazrgar A., Bafrani M.A., Nasab M.M.M., Noroozi M. (2025). Efficacy of orlistat for the treatment of metabolic dysfunction-associated steatotic liver disease: A systematic review and meta-analysis. Hepatol. Forum.

[8] Karimi M., Pourfaraji S.M.A., Parhizkar Roudsari P., Pirzad S. (2026). Efficacy of orlistat on cardiometabolic indices in patients with metabolic dysfunction-associated steatotic liver disease (MASLD): A GRADE-assessed systematic review and meta-analysis of RCTs. Curr. Ther. Res. Clin. Exp..

[9] Grunvald E., Shah R., Hernaez R., Chandar A.K., Pickett-Blakely O., Teigen L.M., Harindhanavudhi T., Sultan S., Singh S., Davitkov P. (2022). AGA clinical practice guideline on pharmacological interventions for adults with obesity. Gastroenterology.

[10] Li W., Liu C., Zhang Z., Cai Z., Lv T., Zhang R., Zuo Y., Chen S. (2024). Exploring the top 30 drugs associated with drug-induced constipation based on the FDA adverse event reporting system. Front. Pharmacol..

[11] U.S. Food and Drug Administration (2022). Xenical (Orlistat) Capsules, for Oral Use: Prescribing Information.

[12] Zhu J., Hu M., Liang Y., Zhong M., Chen Z., Wang Z., Yang Y., Luo Z., Zeng W., Li J. (2024). Pharmacovigilance analysis of orlistat adverse events based on the FDA adverse event reporting system (FAERS) database. Heliyon.

[13] Sakaeda T., Tamon A., Kadoyama K., Okuno Y. (2013). Data mining of the public version of the FDA adverse event reporting system. Int. J. Med. Sci..

[14] Bate A., Evans S.J.W. (2009). Quantitative signal detection using spontaneous ADR reporting. Pharmacoepidemiol. Drug Saf..

[15] U.S. Food and Drug Administration (2026). FDA Adverse Event Monitoring System (AEMS) Public Dashboard and Data Limitations.

[16] U.S. Food and Drug Administration (2007). Alli (Orlistat 60 mg Capsules): Over-the-Counter Label.

[17] U.S. Food and Drug Administration (2010). Xenical (Orlistat) NDA 020766/S-028 Supplement Approval Letter: Labeling Changes Regarding Severe Liver Injury.

[18] Hoffman K.B., Demakas A.R., Dimbil M., Tatonetti N.P., Erdman C.B. (2014). Stimulated reporting: The impact of U.S. Food and Drug Administration-issued alerts on the Adverse Event Reporting System (FAERS). Drug Saf..

[19] European Medicines Agency (2022). Xenical: EPAR Product Information.

[20] Turkish Medicines and Medical Devices Agency (TİTCK) (2020). Xenical 120 mg Sert Kapsül: Kısa Ürün Bilgisi [Turkish Prescribing Information].

[21] Guarino A.H. (2005). Treatment of intractable constipation with orlistat: A report of three cases. Pain Med..

[22] Iqbal F., Samuel M., Tan E.J.K., Nicholls R.J., Vaizey C.J. (2016). Letter: Orlistat as a potential treatment for chronic idiopathic constipation. Aliment. Pharmacol. Ther..

[23] Chukhin E., Takala P., Hakko H., Raidma M., Putkonen H., Räsänen P., Terevnikov V., Stenberg J.-H., Eronen M., Joffe G. (2013). In a randomized placebo-controlled add-on study orlistat significantly reduced clozapine-induced constipation. Int. Clin. Psychopharmacol..

[24] Zabihi M., Naseri F., Ghanei A. (2025). The impact of orlistat on diabetic patients experiencing overweight and constipation: A randomized clinical trial. Iran. J. Diabetes Obes..

[25] Packard K.A., Wurdeman R.L., Reyes A.P. (2002). Constipation, polyuria, polydipsia, and edema associated with orlistat. Ann. Pharmacother..

[26] Van Der Schoot A., Katsirma Z., Whelan K., Dimidi E. (2024). Systematic review and meta-analysis: Foods, drinks and diets and their effect on chronic constipation in adults. Aliment. Pharmacol. Ther..

[27] Levy R.L., Linde J.A., Feld K.A., Crowell M.D., Jeffery R.W. (2005). The association of gastrointestinal symptoms with weight, diet, and exercise in weight-loss program participants. Clin. Gastroenterol. Hepatol..

[28] Ueki T., Nakashima M. (2019). Relationship between constipation and medication. J. UOEH.

[29] Silveira E.A., Santos A.S.E.A.C., Ribeiro J.N., Noll M., Rodrigues A.P.S., de Oliveira C. (2021). Prevalence of constipation in adults with obesity class II and III and associated factors. BMC Gastroenterol..

[30] Eslick G.D. (2012). Gastrointestinal symptoms and obesity: A meta-analysis. Obes. Rev..

[31] Li Z., Maglione M., Tu W., Mojica W., Arterburn D., Shugarman L.R., Hilton L., Suttorp M., Solomon V., Shekelle P.G. (2005). Meta-analysis: Pharmacologic treatment of obesity. Ann. Intern. Med..

[32] Acharya N.V., Wilton L.V., Shakir S.A.W. (2006). Safety profile of orlistat: Results of a prescription-event monitoring study. Int. J. Obes..

[33] Sall D., Wang J., Rashkin M., Welch J., Droege C., Schauer D. (2014). Orlistat-induced fulminant hepatic failure. Clin. Obes..

[34] Zelber-Sagi S., Kessler A., Brazowsky E., Webb M., Lurie Y., Santo M., Leshno M., Blendis L., Halpern Z., Oren R. (2006). A double-blind randomized placebo-controlled trial of orlistat for the treatment of nonalcoholic fatty liver disease. Clin. Gastroenterol. Hepatol..

[35] Stinton L.M., Shaffer E.A. (2012). Epidemiology of gallbladder disease: Cholelithiasis and cancer. Gut Liver.

[36] Zhu Q., Sun X., Ji X., Zhu L., Xu J., Wang C., Zhang C., Xue F., Liu Y. (2016). The association between gallstones and metabolic syndrome in urban Han Chinese: A longitudinal cohort study. Sci. Rep..

[37] Chang Y., Noh Y.-H., Suh B.-S., Kim Y., Sung E., Jung H.-S., Kim C.-W., Kwon M.-J., Yun K.E., Noh J.-W. (2018). Bidirectional association between nonalcoholic fatty liver disease and gallstone disease: A cohort study. J. Clin. Med..

[38] Liu C.-M., Hsu C.-T., Li C.-Y., Chen C.-C., Liu M.-L., Liu J.-H. (2012). A population-based cohort study of symptomatic gallstone disease in diabetic patients. World J. Gastroenterol..

[39] Björkström K., Franzén S., Eliasson B., Miftaraj M., Gudbjörnsdottir S., Trolle-Lagerros Y., Svensson A.-M., Hagström H. (2019). Risk factors for severe liver disease in patients with type 2 diabetes. Clin. Gastroenterol. Hepatol..

[40] Giunchi V., Fusaroli M., Hauben M., Raschi E., Poluzzi E. (2023). Challenges and opportunities in accessing and analysing FAERS data: A call towards a collaborative approach. Drug Saf..

[41] Mathus-Vliegen E.M.H., van Ierland-van Leeuwen M.L., Roolker W., Tytgat G.N.J. (2004). Lipase inhibition by orlistat: Effects on gall-bladder kinetics and cholecystokinin release in obesity. Aliment. Pharmacol. Ther..

[42] Ellrichmann M., Ritter P.R., Otte J.-M., Schrader H., Banasch M., Brunke G., Herzig K.-H., Seebeck J., Schmidt W.E., Schmitz F. (2007). Orlistat reduces gallbladder emptying by inhibition of CCK release in response to a test meal. Regul. Pept..

[43] Napier S., Thomas M. (2006). 36-year-old man presenting with pancreatitis and a history of recent commencement of orlistat: Case report. Nutr. J..

[44] Ahmad F.A., Mahmud S. (2010). Acute pancreatitis following orlistat therapy: Report of two cases. JOP.

[45] Kose M., Emet S., Akpinar T.S., Ilhan M., Gok A.F.K., Dadashov M., Tukek T. (2015). An unexpected result of obesity treatment: Orlistat-related acute pancreatitis. Case Rep. Gastroenterol..

[46] MedDRA MSSO (2024). MedDRA Introductory Guide.

[47] Brown E.G., Wood L., Wood S. (1999). The medical dictionary for regulatory activities (MedDRA). Drug Saf..

[48] Hazell L., Shakir S.A.W. (2006). Under-reporting of adverse drug reactions: A systematic review. Drug Saf..

[49] U.S. Food and Drug Administration (2026). FDA Adverse Event Monitoring System (AEMS): Latest Quarterly Data Files.

[50] U.S. Food and Drug Administration (2026). FDA Launches New Adverse Event Look-Up Tool.

[51] Evans S.J., Waller P.C., Davis S. (2001). Use of proportional reporting ratios (PRRs) for signal generation from spontaneous adverse drug reaction reports. Pharmacoepidemiol. Drug Saf..

[52] van Puijenbroek E.P., Bate A., Leufkens H.G.M., Lindquist M., Orre R., Egberts A.C.G. (2002). A comparison of measures of disproportionality for signal detection in spontaneous reporting systems for adverse drug reactions. Pharmacoepidemiol. Drug Saf..

[53] Benjamini Y., Hochberg Y. (1995). Controlling the false discovery rate: A practical and powerful approach to multiple testing. J. R. Stat. Soc. Ser. B Stat. Methodol..

