# Supplementary material for: Orlistat-Associated Gastrointestinal, Hepatobiliary, Pancreatic, and Anorectal Safety Signals: A FAERS Disproportionality and Regulatory Label Concordance Study
Source: Pharmaceuticals (Basel). 2026 Aug 22;19(9):1325. doi: 10.3390/ph19091325 (PMC13610133; doi:10.3390/ph19091325)
Supplement: Supplementary file 1 [file pharmaceuticals-19-01325-s001.zip › Supplementary_Table_S1_Disproportionality_Results.pdf]

**Supplementary Table S1.** Complete disproportionality analysis results for all 65 screened adverse event preferred terms.

| MedDRA Preferred Term | a | b | c | d | ROR | 95% CI Lower | 95% CI Upper | PRR | Signal criteria met |
|---|---|---|---|---|---|---|---|---|---|
| Rectal Discharge | 1640 | 15044 | 1454 | 15,679,018 | 1175.53 | 1093.44 | 1263.78 | 1060.08 | Yes |
| Steatorrhoea | 1278 | 15406 | 1133 | 15,679,339 | 1147.99 | 1058.12 | 1245.50 | 1060.13 | Yes |
| Change of Bowel Habit | 90 | 16594 | 1797 | 15,678,675 | 47.32 | 38.27 | 58.51 | 47.07 | Yes |
| Faeces Pale | 70 | 16614 | 2057 | 15,678,415 | 32.11 | 25.29 | 40.77 | 31.98 | Yes |
| Faeces Discoloured | 453 | 16231 | 20428 | 15,660,044 | 21.40 | 19.47 | 23.51 | 20.84 | Yes |
| Anal Incontinence | 289 | 16395 | 13036 | 15,667,436 | 21.19 | 18.84 | 23.83 | 20.84 | Yes |
| Defaecation Urgency | 142 | 16542 | 6571 | 15,673,901 | 20.48 | 17.33 | 24.20 | 20.31 | Yes |
| Frequent Bowel Movements | 504 | 16180 | 25266 | 15,655,206 | 19.30 | 17.65 | 21.11 | 18.75 | Yes |
| Flatulence | 923 | 15761 | 53718 | 15,626,754 | 17.04 | 15.93 | 18.21 | 16.15 | Yes |
| Abnormal Faeces | 126 | 16558 | 7769 | 15,672,703 | 15.35 | 12.87 | 18.32 | 15.24 | Yes |
| Faeces Hard | 47 | 16637 | 3729 | 15,676,743 | 11.88 | 8.90 | 15.84 | 11.85 | Yes |
| Rectal Haemorrhage | 407 | 16277 | 40801 | 15,639,671 | 9.58 | 8.68 | 10.58 | 9.38 | Yes |
| Gastrointestinal Sounds Abnormal | 41 | 16643 | 4991 | 15,675,481 | 7.74 | 5.69 | 10.53 | 7.72 | Yes |
| Functional Gastrointestinal Disorder | 32 | 16652 | 3904 | 15,676,568 | 7.72 | 5.45 | 10.93 | 7.70 | Yes |
| Abdominal Pain | 1649 | 15035 | 220602 | 15,459,870 | 7.69 | 7.30 | 8.09 | 7.03 | Yes |
| Rectal Tenesmus | 9 | 16675 | 1174 | 15,679,298 | 7.21 | 3.74 | 13.89 | 7.20 | Yes |
| Gastrointestinal Disorder | 741 | 15943 | 111087 | 15,569,385 | 6.51 | 6.05 | 7.01 | 6.27 | Yes |
| Proctalgia | 39 | 16645 | 6198 | 15,674,274 | 5.93 | 4.32 | 8.12 | 5.91 | Yes |
| Constipation | 1133 | 15551 | 201894 | 15,478,578 | 5.59 | 5.26 | 5.93 | 5.27 | Yes |
| Abdominal Pain Upper | 1088 | 15596 | 195648 | 15,484,824 | 5.52 | 5.19 | 5.87 | 5.23 | Yes |

| MedDRA Preferred Term | a | b | c | d | ROR | 95% CI Lower | 95% CI Upper | PRR | Signal criteria met |
|---|---|---|---|---|---|---|---|---|---|
| Gastrointestinal Pain | 61 | 16623 | 10524 | 15,669,948 | 5.46 | 4.25 | 7.03 | 5.45 | Yes |
| Haematochezia | 302 | 16382 | 52777 | 15,627,695 | 5.46 | 4.87 | 6.12 | 5.38 | Yes |
| Abdominal Distension | 541 | 16143 | 98637 | 15,581,835 | 5.29 | 4.86 | 5.77 | 5.15 | Yes |
| Biliary Colic | 18 | 16666 | 3398 | 15,677,074 | 4.98 | 3.13 | 7.92 | 4.98 | Yes |
| Cholelithiasis | 156 | 16528 | 31520 | 15,648,952 | 4.69 | 4.00 | 5.49 | 4.65 | Yes |
| Cholecystitis | 51 | 16633 | 11371 | 15,669,101 | 4.23 | 3.21 | 5.57 | 4.22 | Yes |
| Gastroenteritis | 62 | 16622 | 14034 | 15,666,438 | 4.16 | 3.24 | 5.35 | 4.15 | Yes |
| Hepatitis | 96 | 16588 | 22648 | 15,657,824 | 4.00 | 3.27 | 4.89 | 3.98 | Yes |
| Anal Pruritus | 7 | 16677 | 2081 | 15,678,391 | 3.16 | 1.51 | 6.64 | 3.16 | Yes |
| Pancreatitis | 155 | 16529 | 48813 | 15,631,659 | 3.00 | 2.56 | 3.52 | 2.98 | Yes |
| Haemorrhoids | 60 | 16624 | 18928 | 15,661,544 | 2.99 | 2.32 | 3.85 | 2.98 | Yes |
| Abdominal Tenderness | 15 | 16669 | 4785 | 15,675,687 | 2.95 | 1.78 | 4.90 | 2.95 | Yes |
| Hepatic Necrosis | 11 | 16673 | 3516 | 15,676,956 | 2.94 | 1.63 | 5.32 | 2.94 | Yes |
| Abdominal Pain Lower | 74 | 16610 | 24486 | 15,655,986 | 2.85 | 2.27 | 3.58 | 2.84 | Yes |
| Diarrhoea | 1558 | 15126 | 602825 | 15,077,647 | 2.58 | 2.45 | 2.71 | 2.43 | Yes |
| Hepatic Failure | 69 | 16615 | 28344 | 15,652,128 | 2.29 | 1.81 | 2.91 | 2.29 | Yes |
| Gastrointestinal Infection | 22 | 16662 | 9034 | 15,671,438 | 2.29 | 1.51 | 3.48 | 2.29 | Yes |
| Irritable Bowel Syndrome | 41 | 16643 | 19144 | 15,661,328 | 2.02 | 1.48 | 2.74 | 2.01 | Yes |
| Pancreatitis Acute | 44 | 16640 | 21332 | 15,659,140 | 1.94 | 1.44 | 2.61 | 1.94 | No |
| Jaundice | 51 | 16633 | 25881 | 15,654,591 | 1.85 | 1.41 | 2.44 | 1.85 | No |
| Abdominal Discomfort | 298 | 16386 | 161027 | 15,519,445 | 1.75 | 1.56 | 1.97 | 1.74 | No |
| Diarrhoea Haemorrhagic | 17 | 16667 | 9070 | 15,671,402 | 1.76 | 1.09 | 2.84 | 1.76 | No |
| Colitis | 72 | 16612 | 40729 | 15,639,743 | 1.66 | 1.32 | 2.10 | 1.66 | No |
| Dyspepsia | 154 | 16530 | 93468 | 15,587,004 | 1.55 | 1.33 | 1.82 | 1.55 | No |
| Gastric Ulcer | 26 | 16658 | 18351 | 15,662,121 | 1.33 | 0.91 | 1.96 | 1.33 | No |

| MedDRA Preferred Term | a | b | c | d | ROR | 95% CI Lower | 95% CI Upper | PRR | Signal criteria met |
|---|---|---|---|---|---|---|---|---|---|
| Eructation | 25 | 16659 | 18162 | 15,662,310 | 1.29 | 0.87 | 1.92 | 1.29 | No |
| Liver Disorder | 53 | 16631 | 41477 | 15,638,995 | 1.20 | 0.92 | 1.57 | 1.20 | No |
| Gastritis | 32 | 16652 | 25516 | 15,654,956 | 1.18 | 0.83 | 1.67 | 1.18 | No |
| Vomiting | 478 | 16206 | 445735 | 15,234,737 | 1.01 | 0.92 | 1.10 | 1.01 | No |
| Gastric Ulcer Haemorrhage | 5 | 16679 | 5418 | 15,675,054 | 0.87 | 0.36 | 2.08 | 0.87 | No |
| Nausea | 649 | 16035 | 736363 | 14,944,109 | 0.82 | 0.76 | 0.89 | 0.83 | No |
| Regurgitation | 3 | 16681 | 3423 | 15,677,049 | 0.82 | 0.27 | 2.56 | 0.82 | No |
| Gastrointestinal Motility Disorder | 4 | 16680 | 5111 | 15,675,361 | 0.74 | 0.28 | 1.96 | 0.74 | No |
| Cholestasis | 14 | 16670 | 18282 | 15,662,190 | 0.72 | 0.43 | 1.22 | 0.72 | No |
| Haematemesis | 18 | 16666 | 24293 | 15,656,179 | 0.70 | 0.44 | 1.11 | 0.70 | No |
| Retching | 15 | 16669 | 20654 | 15,659,818 | 0.68 | 0.41 | 1.13 | 0.68 | No |
| Colitis Ischaemic | 4 | 16680 | 6448 | 15,674,024 | 0.58 | 0.22 | 1.55 | 0.58 | No |
| Gastrointestinal Inflammation | 4 | 16680 | 7510 | 15,672,962 | 0.50 | 0.19 | 1.33 | 0.50 | No |
| Gastrooesophageal Reflux Disease | 40 | 16644 | 76115 | 15,604,357 | 0.49 | 0.36 | 0.67 | 0.49 | No |
| Melaena | 11 | 16673 | 21315 | 15,659,157 | 0.48 | 0.27 | 0.88 | 0.49 | No |
| Gastrointestinal Haemorrhage | 37 | 16647 | 80726 | 15,599,746 | 0.43 | 0.31 | 0.59 | 0.43 | No |
| Dry Mouth | 27 | 16657 | 77333 | 15,603,139 | 0.33 | 0.22 | 0.48 | 0.33 | No |
| Dysphagia | 26 | 16658 | 92152 | 15,588,320 | 0.26 | 0.18 | 0.39 | 0.27 | No |
| Mouth Ulceration | 5 | 16679 | 20081 | 15,660,391 | 0.23 | 0.10 | 0.56 | 0.23 | No |
| Oesophagitis | 1 | 16683 | 10510 | 15,669,962 | 0.09 | 0.01 | 0.63 | 0.09 | No |

Complete disproportionality analysis results for all 65 MedDRA preferred terms representing gastrointestinal, hepatobiliary, pancreatic, and anorectal adverse events evaluated in association with orlistat in the FAERS database (2004 Q1–2026 Q1). Candidate adverse-event concepts were identified based on the pharmacological mechanism of orlistat, adverse reactions and post-marketing events described in the Turkish, European Union, and United States prescribing information, and relevant clinical and pharmacovigilance literature. These concepts were mapped to MedDRA version 27.0 preferred terms and grouped into four predefined clinical domains. The final list of 65 MedDRA preferred terms was completed before calculation of term-specific RORs, PRRs, and confidence intervals or determination of signal status. a = primary-suspect orlistat reports with the target adverse event; b = primary-suspect orlistat

reports without the target adverse event; c = non-orlistat reports with the target adverse event; d = non-orlistat reports without the target adverse event; CI = confidence interval; FAERS = FDA Adverse Event Reporting System; MedDRA = Medical Dictionary for Regulatory Activities; PRR = proportional reporting ratio; ROR = reporting odds ratio. Signal threshold: ROR > 2, lower bound of the 95% CI > 1, and at least three cases (a ≥ 3). Total primary-suspect orlistat reports (a + b) = 16,684; total non-orlistat reports (c + d) = 15,680,472.
