# Supplementary material for: Orlistat-Associated Gastrointestinal, Hepatobiliary, Pancreatic, and Anorectal Safety Signals: A FAERS Disproportionality and Regulatory Label Concordance Study
Source: Pharmaceuticals (Basel). 2026 Aug 22;19(9):1325. doi: 10.3390/ph19091325 (PMC13610133; doi:10.3390/ph19091325)
Supplement: Supplementary file 1 [file pharmaceuticals-19-01325-s001.zip › Supplementary_Table_S2_FDR_Adjusted_Results.pdf]

Supplementary Table S2. Unadjusted p values and Benjamini–Hochberg false discovery rate–adjusted q values for the 65 prespecified MedDRA preferred terms

MedDRA preferred term	Unadjusted *p* value	BH-adjusted *q* value
Rectal Discharge	<0.001	<0.001
Steatorrhoea	<0.001	<0.001
Change of Bowel Habit	<0.001	<0.001
Faeces Pale	<0.001	<0.001
Faeces Discoloured	<0.001	<0.001
Anal Incontinence	<0.001	<0.001
Defaecation Urgency	<0.001	<0.001
Frequent Bowel Movements	<0.001	<0.001
Flatulence	<0.001	<0.001
Abnormal Faeces	<0.001	<0.001
Faeces Hard	<0.001	<0.001
Rectal Haemorrhage	<0.001	<0.001
Gastrointestinal Sounds Abnormal	<0.001	<0.001
Functional Gastrointestinal Disorder	<0.001	<0.001
Abdominal Pain	<0.001	<0.001
Rectal Tenesmus	<0.001	<0.001
Gastrointestinal Disorder	<0.001	<0.001
Proctalgia	<0.001	<0.001
Constipation	<0.001	<0.001
Abdominal Pain Upper	<0.001	<0.001
Gastrointestinal Pain	<0.001	<0.001
Haematochezia	<0.001	<0.001
Abdominal Distension	<0.001	<0.001
Biliary Colic	<0.001	<0.001
Cholelithiasis	<0.001	<0.001
Cholecystitis	<0.001	<0.001
Gastroenteritis	<0.001	<0.001
Hepatitis	<0.001	<0.001
Anal Pruritus	0.008	0.010
Pancreatitis	<0.001	<0.001
Haemorrhoids	<0.001	<0.001
Abdominal Tenderness	<0.001	<0.001
Hepatic Necrosis	0.002	0.002
Abdominal Pain Lower	<0.001	<0.001
Diarrhoea	<0.001	<0.001
Hepatic Failure	<0.001	<0.001
Gastrointestinal Infection	<0.001	<0.001
Irritable Bowel Syndrome	<0.001	<0.001
Pancreatitis Acute	<0.001	<0.001

MedDRA preferred term	Unadjusted *p* value	BH-adjusted *q* value
Jaundice	<0.001	<0.001
Abdominal Discomfort	<0.001	<0.001
Diarrhoea Haemorrhagic	0.033	0.042
Colitis	<0.001	<0.001
Dyspepsia	<0.001	<0.001
Gastric Ulcer	0.140	0.169
Eructation	0.208	0.236
Liver Disorder	0.175	0.203
Gastritis	0.336	0.364
Vomiting	0.852	0.879
Gastric Ulcer Haemorrhage	1.000	1.000
Nausea	<0.001	<0.001
Regurgitation	1.000	1.000
Gastrointestinal Motility Disorder	0.828	0.868
Cholestasis	0.255	0.281
Haematemesis	0.139	0.169
Retching	0.163	0.193
Colitis Ischaemic	0.341	0.364
Gastrointestinal Inflammation	0.211	0.236
Gastrooesophageal Reflux Disease	<0.001	<0.001
Melaena	0.011	0.014
Gastrointestinal Haemorrhage	<0.001	<0.001
Dry Mouth	<0.001	<0.001
Dysphagia	<0.001	<0.001
Mouth Ulceration	<0.001	<0.001
Oesophagitis	<0.001	<0.001

Two-sided p values were calculated with Fisher's exact test applied to the same 2 × 2 contingency tables used for the reporting odds ratio analyses and were adjusted across all 65 prespecified MedDRA preferred terms using the Benjamini–Hochberg false discovery rate procedure. Fisher's exact test was used because the expected cell count was below 5 for 11 of the 65 preferred terms. A BH-adjusted q value < 0.05 was considered statistically significant. FDR adjustment was used as a complementary robustness assessment and did not replace the primary pharmacovigilance signal definition of ROR > 2.0, lower bound of the 95% confidence interval > 1.0, and $n \geq 3$. Values below 0.001 are reported as <0.001. MedDRA, Medical Dictionary for Regulatory Activities; PT, preferred term; ROR, reporting odds ratio.
