# Supplementary material for: Orlistat-Associated Gastrointestinal, Hepatobiliary, Pancreatic, and Anorectal Safety Signals: A FAERS Disproportionality and Regulatory Label Concordance Study
Source: Pharmaceuticals (Basel). 2026 Aug 22;19(9):1325. doi: 10.3390/ph19091325 (PMC13610133; doi:10.3390/ph19091325)
Supplement: Supplementary file 1 [file pharmaceuticals-19-01325-s001.zip › Supplementary_Table_S3_Drug_Name_Normalisation.pdf]

Supplementary Table S3. Drug-name normalisation rules and case-definition criteria used to identify orlistat-associated reports in the FAERS database (2004 Q1–2026 Q1).

Entry or condition	Classification	Case-definition decision	Rationale
ORLISTAT	Exact active-ingredient identifier	Include	An exact ORLISTAT match in the active-ingredient field was required for inclusion.
ALLI	Recognised brand name	Include only with an ORLISTAT active-ingredient match	Brand-name information alone was not sufficient to meet the case definition.
XENICAL	Recognised brand name	Include only with an ORLISTAT active-ingredient match	Brand-name information alone was not sufficient to meet the case definition.
No exact ORLISTAT match	Exclusion condition	Exclude	Records without an explicit ORLISTAT active-ingredient match did not meet the case definition.
Missing, unspecified, or alternative product name	Non-confirmatory product-name information	Include only with an ORLISTAT active-ingredient match	Missing or alternative product-name information did not lead to exclusion when the exact active ingredient was documented.
Additional spelling variants	None identified	No additional variants included	No additional spelling variants were observed in the analytical dataset.
Differences in letter case or leading/trailing spaces	Formatting variation	Normalise before matching	Entries were trimmed and matched case-insensitively.
Multiple qualifying entries within one deduplicated CASEID	Repeated qualifying drug entries	Count once	Each deduplicated CASEID contributed only one report to the analytical population.

Note: Drug-name normalisation used the FAERS “Suspect Product Names” and “Suspect Product Active Ingredients” fields. ORLISTAT was the exact active-ingredient identifier. ALLI and XENICAL were the only recognised brand names observed. Product-name information alone was not sufficient for inclusion. Records without an exact ORLISTAT active-ingredient match were excluded, and multiple qualifying entries within the same deduplicated CASEID were counted once. The 16,684 reports in which orlistat had the primary-suspect role were retained for the disproportionality analyses.

Abbreviations: CASEID, FAERS case identifier; FAERS, FDA Adverse Event Reporting System.
